# Supplementary material for: Reversible p53 inhibition prevents cisplatin ototoxicity without blocking chemotherapeutic efficacy
Source: EMBO Mol Med. 2016 Oct 28;9(1):7–26. doi: 10.15252/emmm.201606230 (PMC5210089; doi:10.15252/emmm.201606230)
Supplement: Supplementary file 1 — Expanded View Figures PDF [file EMMM-9-7-s001.pdf]

Expanded View Figures

Figure EV1. Cochlear explant and slice preparation, and CDDP-induced DNA damage and cell death in sensory hair cells and spiral ganglion neurons.

A, B Representative images showing an organ of Corti explant and a cochlear slice (250–300 μm thickness) cultured with culture medium alone for 5 days. The explant and slice were then immunolabeled with myosin 7A (red) and NF200 (green). SG: spiral ganglion, nf: nerve fiber, SV: scala vestibuli, SM: scala media, ST: scala tympani, oC: organ of Corti. Scale bars = (A), 300 μm; (B), 60 μm.

C Confocal images showing the spiral ganglion neurons from the basal turns of cochlear slices treated with either culture medium alone or medium containing 10 μM CDDP for 3 days before being cultured for a further 2 days. The slices were labeled with NF200 (green) to highlight auditory nerve fibers and neurons and Hoechst 33342 dye (blue) to label chromatin. Scale bar = 20 μm.

D Quantification analysis of spiral ganglion neuron density in control and CDDP-exposed cochlear slices (n = 2–3 slices per cochlea and 5 cochleae per group, all experiments were performed in triplicate). Data are expressed as mean ± SEM. One-way ANOVA test followed by *post hoc* Tukey's test ($P > 0.05$).

E–G Confocal images showing the basal region of cochlear explants treated with culture medium either alone (E) or containing 10 μM CDDP for 3 days (F, G). Hair cells were identified using myosin 7A (red), phosphatidylserine sites on the cell membrane surface using fluorochrome-labeled Annexin V (green in F), and apoptotic DNA fragmentation using a TUNEL apoptosis kit (green in G). The white arrowheads indicate cell surface Annexin V-positive labeling and TUNEL-positive nuclei in some CDDP-damaged IHCs. Scale bar = 20 μm.

H, I Confocal images showing the spiral ganglion neurons from the basal turn of cochlear slice cultures treated with culture medium either alone or containing 10 μM CDDP for 1 day and immunolabeled for NF200 (red) and γH2AX (green in H), or NF200 (red) and 53BP1 (green in I). The white arrowheads indicate CDDP-induced γH2AX and 53BP1 foci in some spiral ganglion neurons. Scale bar = 20 μm.

▶

© 2016 The Authors

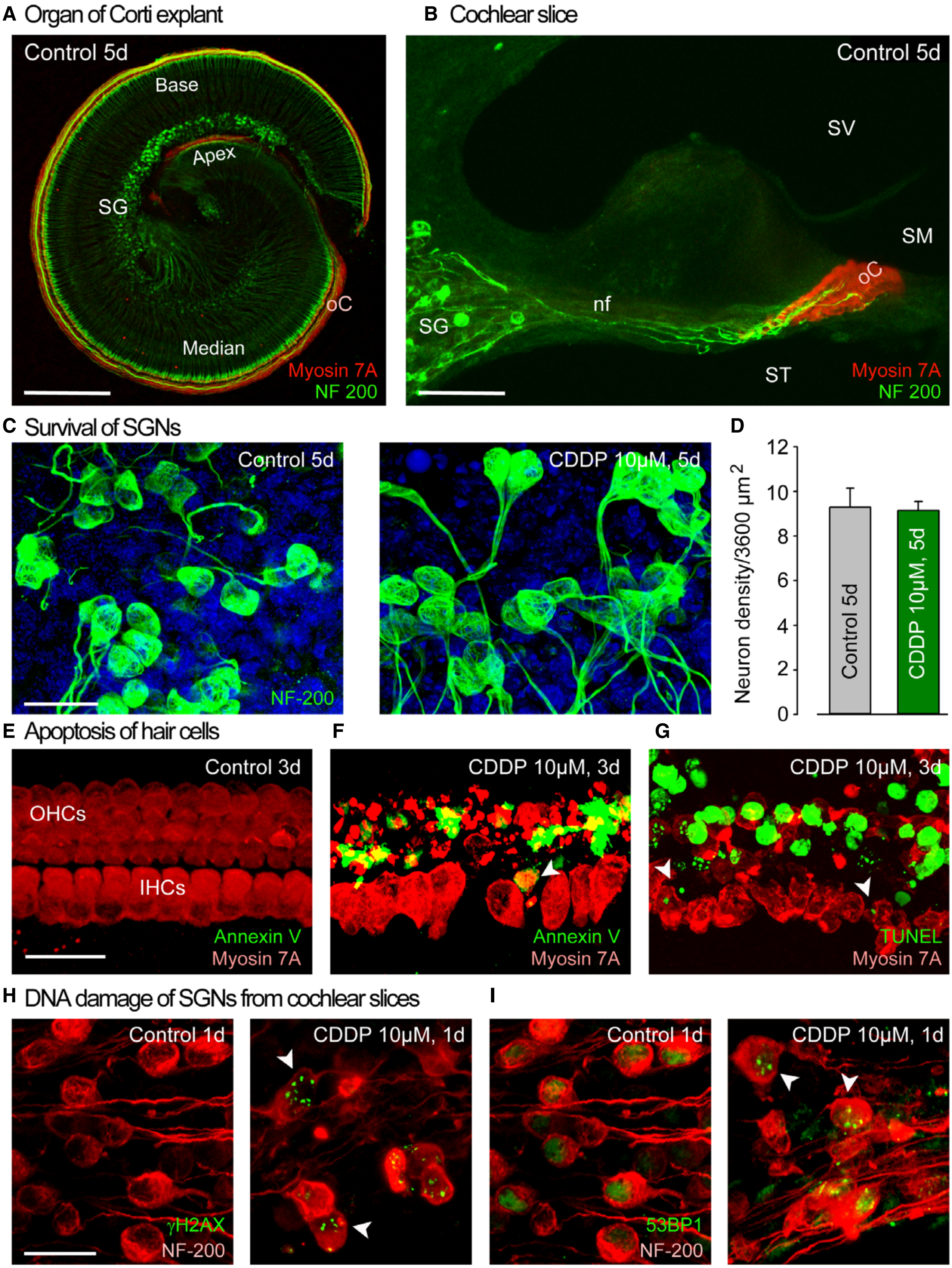

Figure EV1.

 © 2016 The Authors

Figure EV2. Effects of CDDP treatment on the DNA damage pathways, and ATM and p53 inhibition.

- A Representative PCR analysis of mATR and mChk1 expression in cochleae, testis, and kidney tissues from P3 mice. Water was used as a negative control.
- B Representative Western blot analysis showing the level of Chk1 phosphorylation in protein extracts from cultured whole cochleae treated or not with 10 μM CDDP and from control and UV light-exposed mouse embryonic fibroblasts (MEF).
- C, D 3D images showing IHC and OHC nuclei from the basal region of the organ of Corti cultures treated with medium alone (control) or 10 μM CDDP for 1 day and immunolabeled for p-ATM (green in C) or p-Chk2 (green in D). Scale bar = 5 μm.
- E 3D images showing OHC nuclei from the basal region of the organ of Corti cultures treated with medium alone, 10 μM KU55933, 10 μM CDDP, or 10 μM CDDP in combination with 10 μM KU55933 for 1 day and immunolabeled for p-ATM (green). Scale bar = 5 μm.
- F Quantification of p-ATM foci number per nucleus in both inner (red bars) and outer (blue bars) hair cells for all treatment conditions (n = 50 nuclei per condition and per time point). Data are expressed as mean ± SEM. One-way ANOVA test followed by *post hoc* Tukey's test (**$P \leq 0.008$, ***$P \leq 0.0006$; CDDP versus control or CDDP versus CDDP + KU55933).
- G Confocal images showing the basal region of organ of Corti cultures treated with medium alone, 100 μM PFT-α, 10 μM CDDP, or 10 μM CDDP in combination with 100 μM PFT-α for 5 days and immunolabeled for myosin 7A (red). Scale bar = 24 μm.
- H Histograms representing the levels of surviving IHCs (red bars) and OHCs (blue bars) for all conditions after 5 days (n = 5 cochleae per condition and per time point). Data are expressed as mean ± SEM. One-way ANOVA test followed by *post hoc* Tukey's test (*$P = 0.02$, **$P \leq 0.006$, ***$P = 0.0004$; CDDP versus control, or CDDP versus CDDP + PFT-α).

Data information: All experiments were performed in triplicate.

© 2016 The Authors

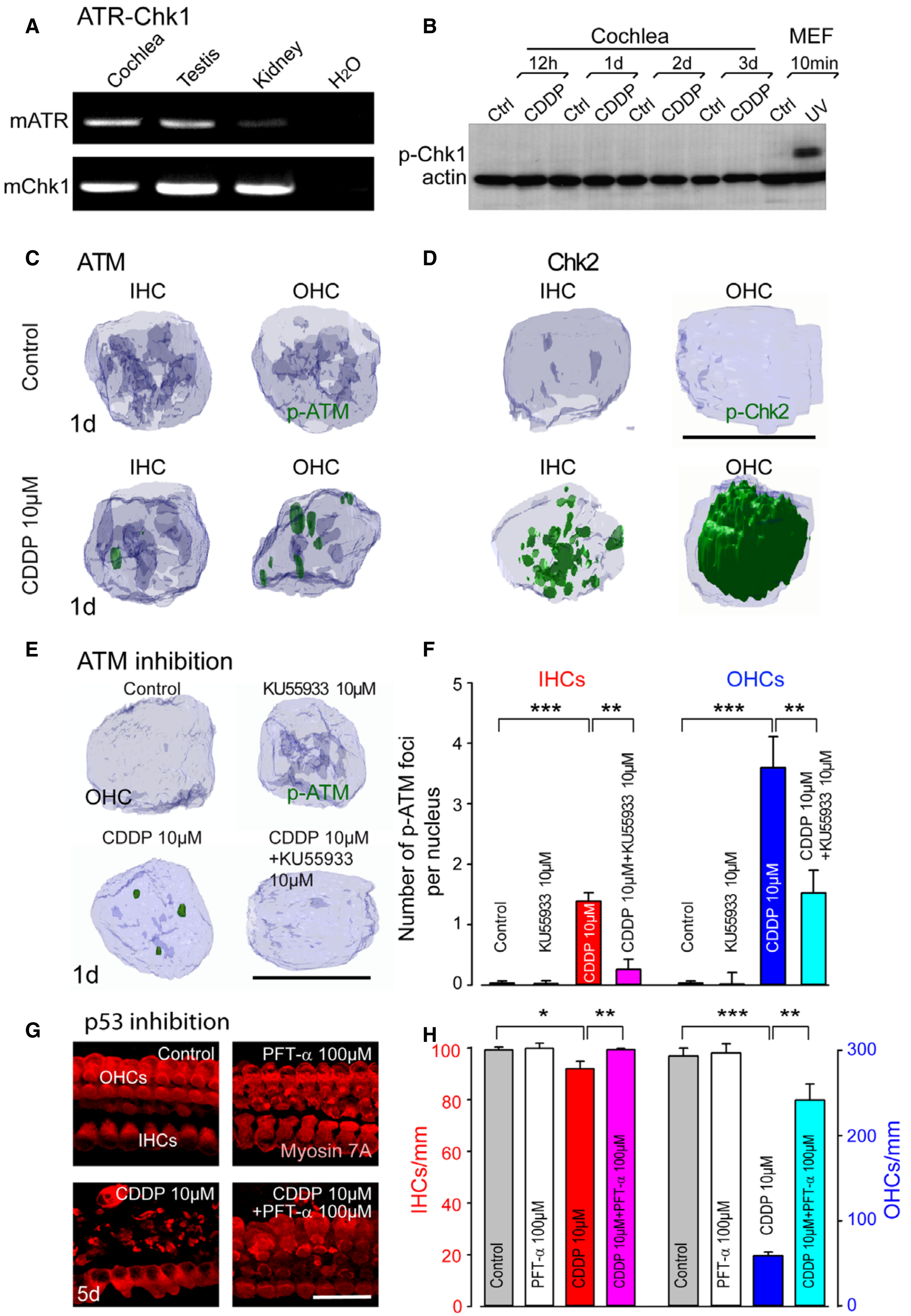

Figure EV2.

 © 2016 The Authors

Figure EV3. Intratympanic injection, tumor histology, and p53 genotyping and activation *in vivo*.

A Shown is the custom-made microendoscope. It consists of a fiber optic lens (10,000-pixel resolution) for viewing and one catheter channel equipped with a fine needle for drug delivery. The blue arrowhead indicates the needle.

B Endoscopic view of the tympanic membrane (left image) and intratympanic injection through a fine needle (right image). The blue arrowhead indicates the needle.

C Representative scanned images of hematoxylin–eosin–safranin-stained *TP53*-mutant (HBCx-14) tumor sections. Tumors were collected at day 21. Right panels: Higher magnification images show tumor features from the black boxed area in the left panels. The black arrowhead in the lower right panel indicates a remaining tumor cell. fs: fibrous scar, tc: tumor cells. Scale bars = left panels, 200 μm; right panels, 50 μm.

D Ratio of fibrous scar area/tumor cell area (sum of fibrous scar area/sum of tumor cell area). Note that combined treatments were much more efficient in replacing tumor cells by fibrous scar. The tumors were collected at day 21 (n = 4 sections/tumor and 3–4 tumors/group). Data are expressed as mean ± SEM. One-way ANOVA test followed by *post hoc* Tukey's test (**P = 0.002, ***P = 0.00008; CDDP versus DMSO or CDDP versus CDDP + PFT-α).

E Representative PCR analysis of p53 expression from p53 wild-type (wt), heterozygous (+/−), and knockout (−/−) mice. The 706-bp band (neomycin cassette) is detected in $p53^{+/-}$ and $p53^{-/-}$, although not in p53wt mice. By contrast, the 470-bp band (p53 wild-type gene) was only detected in p53wt and $p53^{+/-}$ mice.

F Representative Western blots using antibodies against p-p53 (serine 15) and actin in whole cochlear extracts from p53wt mice treated with CDDP for 0, 2, and 5 days. Note the increase in p53 phosphorylation in 2- and 5-day CDDP-treated cochleae.

© 2016 The Authors

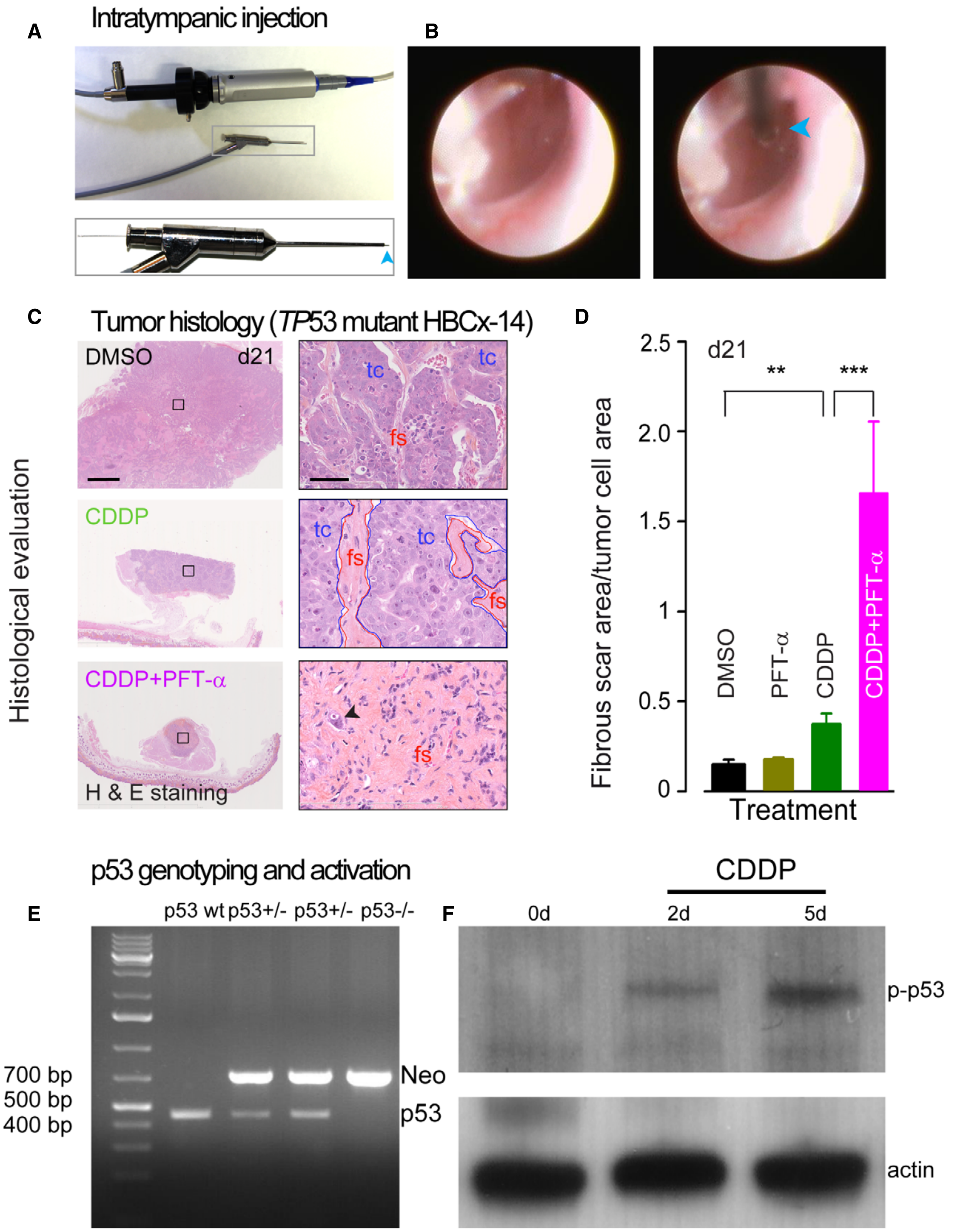

Figure EV3.

 © 2016 The Authors

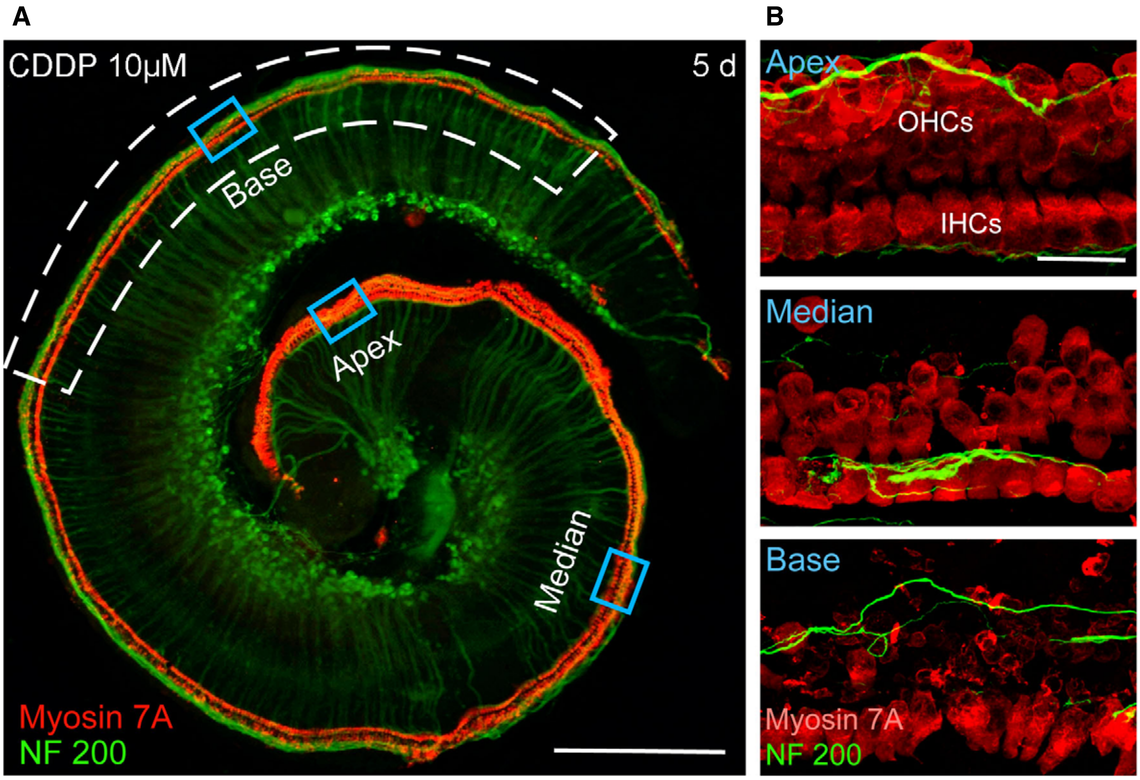

Figure EV4. CDDP-induced OHC loss along a basal-apical gradient.

A, B Representative images showing an organ of Corti explant treated with 10 μM CDDP for 3 days and cultured for a further 2 days. The explant was then immunolabeled with myosin 7A (red) and NF200 (green). Note the OHC loss follows a base-to-apex gradient. The white box in (A) indicates the region in which the observations and counting were performed. (B)Higher magnification images show OHC loss in the base, median, and apex regions indicated by blue boxes in (A). Scale bars = (A), 300 μm; (B), 16 μm.
